# Numerical Investigation of Fluid Flow Through Glass Sphere Packed Bed Representative of High-Porosity Permeable Concrete

**DOI:** 10.1007/s11242-026-02294-5

**Published:** 2026-02-12

**Authors:** Mahmoud Abbaszadeh, Marios Christou, Alalea Kia

**Affiliations:** Department of Civil and Environmental Engineering, Imperial College London, London, SW7 2AZ UK

**Keywords:** Permeable concrete, Porous media, Resolved computational fluid dynamics, Discrete element method, Hydraulic tortuosity, Permeability

## Abstract

Permeable concrete allows rapid drainage of stormwater through otherwise impermeable infrastructure. Modelling the hydraulic performance of permeable concrete is challenging due to its tortuous pore structure. In this study a robust numerical model, validated against experimental results, is developed for permeable concrete structures at higher porosity using glass spheres (GS) of different diameters. Monosized GS along with polysized combinations are used to represent an idealised permeable concrete structure. The effect of the pore characteristics, including porosity and mean pore size, along with the hydraulic gradient (HG) on permeability and hydraulic tortuosity is determined. Discrete element method and computational fluid dynamics (CFD) are both used to generate the GS and simulate water flow through the packed bed, respectively. Permeability measurements are conducted using both falling head (FH) and constant head methods. The results demonstrate a strong agreement between the FH permeability values obtained through the resolved CFD simulations and the experimental data, without requiring any calibration of the CFD model. In summary, the present study offers a high-resolution, experimentally validated and physics-based approach to understanding the permeability and tortuosity of permeable concretes modelled as packed sphere beds. It eliminates reliance on empirical adjustments, provides new insights into the effect of the different HG and establishes a generalised permeability correlation applicable to a wide range of scenarios.

## Introduction

Permeable concrete is used to reduce surface water flooding as it allows stormwater to flow through its porous structure (Scholz and Grabowiecki 2007; Kia et al. 2017; Kia 2023). Permeable concrete has many benefits, including improving stormwater and groundwater quality by capturing pollutants, enhancing skid resistance and minimising heat island effects in cities (Kia et al. 2017; Kia 2022; Debnath and Sarkar 2020; Singh et al. 2019, 2020; Pilon et al. 2019; Braswell et al. 2018). Permeable concrete is difficult to model accurately as it is composed of complex geometries, including curved surfaces, irregular shapes and intricate internal features (Zhang et al. 2018b). It is therefore challenging to create an accurate three-dimensional (3D) model of permeable concrete structure to use in a Computational Fluid Dynamics (CFD) simulation. Alternative modelling approaches have been explored to better understand and predict the permeable concrete behaviour, including through the use of packed spheres to approximate permeable concrete properties (Nan et al. 2021; Zhang et al. 2021, 2018a; Zhu et al. 2022).

Graton and Fraser (1935) examined six different geometric packing arrangements for spheres and highlighted the large changes in permeability simply produced by variation in the packing technique. Generally, the type of packing can be classified into two types, namely structured and unstructured (random), that can be used to create a packed bed column (Amini and Nasr Esfahany 2019; Khirevich 2011), and they can be formed of monosized (single diameter) or polysized (multiple diameters) spheres. Fand et al. (1987) performed experimental investigations on packed beds with both monosized and polysized spheres and identified the Reynolds numbers corresponding to Darcy, Forchheimer and turbulent flow regimes. In the structured method for packed spheres, the position of each sphere can be defined using simple mathematical equations and a suitable coordinate system, by employing computer-aided design (CAD) software or a mesh generation program (Nijemeisland and Dixon 2004; Reddy and Joshi 2008). Since a structured bed is repetitive, its geometry remains consistent throughout the bed and the flow characteristics are unlikely to vary. Whilst the structured packing method can achieve the lowest porosity of 26% for packed spheres (Hales 2008), this method is not suitable for highly disordered geometries where the flow patterns vary significantly across the bed (Manjhi et al. 2006). Conversely, for the unstructured packing approach, a nondeterministic algorithm for sphere packing is necessary to generate the position of each sphere (Zeiser et al. 2001; Jafari et al. 2008). These sphere positions can then be utilised alongside a CAD software and a suitable meshing program to create a functional computational domain. However, this approach has some limitations when simulating permeable concretes with low porosity ($\leqslant \,30\%$). For example, Vasseur et al. (2021) created a numerically generated random packing of spherical particles with porosity greater than 38%. Xu et al. (2022) used a relaxation iteration scheme to develop random packing of monodisperse and polydisperse spheroidal particles but achieved porosity values in excess of 62%. To achieve low porosity in the packing process, Zhang et al. (2018a) considered the spheres to have a low shear modulus resulting in their overlap. However, overlapping spheres are impossible to achieve in experiments since physical properties cannot be modified. In this paper, the unstructured bed method without overlap is used to investigate the influence of random packing on the hydraulic properties of glass spheres (GS), with the results validated against the experimental measurements in Kia et al. (2018).

There are four primary hydraulic properties associated with GS: (i) porosity; (ii) pore size distribution; (iii) permeability; and (iv) tortuosity. Porosity is typically determined by measuring the void volume in GS and comparing it to its total packed bed volume (Kia et al. 2018), whilst pore size distribution refers to a range of different pore sizes within GS and can be measured by image analysis (Neithalath et al. 2010b). The permeability is a measure of how easily a fluid can flow through GS. Although the terms permeability and hydraulic conductivity have been used interchangeably in concrete science and hydrology, their units differ. Permeability is typically measured in Darcy (*d*), where 1 Darcy equals $10^{-12}$ m^2^. However, for permeability of permeable concrete and GS, the unit that is commonly used is ${\text {m}}\,{\text {s}}^{-1}$, which is the same as the unit used for hydraulic conductivity (Kia et al. 2018). To convert hydraulic conductivity in ${\text {m}}\,{\text {s}}^{-1}$ to permeability in m^2^, the dynamic viscosity $\mu$ (Pas) and density of the fluid $\rho$ (kg m$^{-3}$) as well as the acceleration due to gravity *g* (m s$^{-2}$) need to be incorporated. In this paper, the term permeability is used throughout and is considered to be equivalent to hydraulic conductivity. Permeability can be determined using falling head (FH) and/or constant head (CH) methods. In a FH method, the hydraulic head decreases over time and the time that is required for the head to drop by a certain predetermined amount is measured (Das 2021; Sumanasooriya et al. 2009; Montes and Haselbach 2006; Neithalath et al. 2010a). In a CH method, a constant hydraulic head is maintained across the sample and the volumetric flow rate is measured (Haselbach et al. 2006). A small number of studies compared the experimental permeability results obtained using FH and CH methods (Zhang et al. 2020; Qin et al. 2015). Zhang et al. (2020) investigated the effect of aggregate gradation and cement-to-aggregate ratio on the permeability of permeable concrete measured using both FH and CH methods. The permeability values obtained using the FH method were found to be higher than those measured using the CH method. This discrepancy was attributed to the upper and lower cross sections of the tube being the same in the FH method, resulting in smooth flow of water down the permeable tube, whilst in the CH method the small diameter of the overflow part was found to hinder water discharge, consequently impacting the permeability test results (Zhang et al. 2020). Furthermore, a linear correlation between the permeability values obtained from FH and CH methods was determined for permeable concrete samples of different porosity, applicable only to the permeable concrete samples of different porosity tested in that particular study (Zhang et al. 2020). Qin et al. (2015) used FH and CH methods to measure the permeability of two permeable concrete mixtures subjected to different hydraulic gradients (HGs). The FH method was found to result in higher permeability values than those measured using the CH method. This difference in the permeability results was attributed to the FH method assuming that the permeability values remain unaffected by the applied pressure, along with the significant effect of the chosen initial and final water heads on the calculated permeability (Qin et al. 2015). It has therefore been suggested that the permeability values should be reported together with the applied pressure and the testing method used, to ensure accurate representation and comparison of the results. Batezini and Balbo (2015) measured the permeability of three different permeable concrete mixtures of varying coarse aggregate sizes, using both FH and CH methods. The FH method was found to result in higher permeability values compared with the CH method and this was believed to be due to the limitations in the FH permeameter not providing the required lateral confinement to the samples. Akkaya and Çağatay (2021) compared the FH and CH permeability results obtained for permeable concrete mixtures prepared with different aggregate sizes and aggregate/binder ratios. For a fixed HG, a strong linear correlation was found between the permeability results of different concrete mixtures used in this study, obtained using FH and CH methods. The permeability values obtained using the CH method were found to be lower than that of the FH method. An increase in the aggregate size was shown to increase permeability, whilst resulting in the difference between the permeability values measured using the FH and CH methods to become more pronounced. It is worth mentioning that Akkaya and Çağatay (2021) did not explicitly elaborate on the specific reasons behind their findings. Sandoval et al. (2017) used four different aggregate types to prepare sustainable permeable concrete mixtures and determined the permeability values of these mixtures using both FH and CH test methods. The permeability values obtained using the FH method were found to be significantly lower than that obtained using the CH method. These results are in contrast with the findings of Zhang et al. (2020), Qin et al. (2015), Batezini and Balbo (2015), and Akkaya and Çağatay (2021) and no explanation has been provided for the reasons behind these observed differences. The correlation between the permeability values obtained from FH and CH methods was determined using a power law equation, that is only valid for the materials and the experimental results obtained in this study. In general, whilst the literature shows the permeability values obtained using the FH method to be higher than that of the CH method, there is a lack of specific information regarding the effect of different parameters/conditions (e.g. HG) that lead to the permeability obtained from the FH method to surpass that of the CH method. Furthermore, the correlations defined between the permeability values of both FH and CH methods are not general and can only be applied to the specific materials and experimental results obtained in those particular studies.

Tortuosity is another hydraulic property and is defined as a geometric parameter or one that is related to hydraulic, diffusive or electrical properties (Ghanbarian et al. 2013; Fu et al. 2021; Duda et al. 2011; Abderrahmene et al. 2017). The present study focuses exclusively on hydraulic tortuosity, as this definition establishes a correlation between the velocity field within the GS and the geometric configuration of the packed bed. Hydraulic tortuosity is defined as the mean trajectory that fluid parcels take as they flow through a permeable medium. According to Clennell (1997), the hydraulic tortuosity should be calculated as a kinematic average, taking into account the weighting of streamlines with fluid fluxes. Due to the difficulties in averaging, previous attempts have focused on simpler models where the results were independent of the specific averaging method used (Duda et al. 2011). Duda et al. (2011) introduced an effective method for calculating hydraulic tortuosity in any geometric configuration, without relying on streamlines, by deriving tortuosity directly from the velocity of the flow field. In this paper, a similar approach is adopted and the hydraulic tortuosity is calculated by deriving it from the CFD data as opposed to conducting experiments (see Sect. 2.3). This is due to the inherent difficulties in the experimental determination of hydraulic tortuosity (Fu et al. 2021), with flow velocity measurements inside a laboratory specimen presenting significant challenges, whilst numerical modelling allows for the necessary flow velocity components within the specimen to be obtained.

 CFD and discrete element method (DEM) have been extensively utilised to analyse the permeability of GS and permeable concretes (Nan et al. 2021; Dixon and Partopour 2020; Zhang et al. 2018a, 2022). CFD is used to model the continuous fluid phase, while DEM is used to model the discrete particle phase (Nan et al. 2021). In CFD simulation of GS, two main methods, namely resolved and unresolved, can be used for meshing the flow domain. In the resolved method, the size of the fluid cells in the computational domain is much smaller than the radius of the GS, while in the unresolved method, the size of the fluid cells is larger than the radius of the GS (Norouzi et al. 2016). In other words, in the resolved approach, fluid flow is completely resolved across the entire surface of GS, resulting in detailed velocity profiles around all GS (see Fig. 2a). These profiles are then combined to determine the total hydrodynamic forces exerted on individual GS. This approach is computationally expensive, as it requires a very fine mesh to fully resolve the flow in the entire domain (Norouzi et al. 2016) (see Fig. 2a). Conversely, the unresolved method utilises fluid cells larger than the GS radius, enabling the study of systems with hundreds of thousand GS. The coupling between the GS and the fluid phase occurs through drag forces and empirical or theoretical correlations, designed to describe the flow across GS (Norouzi et al. 2016). While there is a substantial body of literature on obtaining permeability using unresolved CFD with CH conditions (Lin et al. 2022; AlShareedah and Nassiri 2021; Nan et al. 2021; Zhang et al. 2018a, 2022), there are very few studies that specifically determine the permeability using resolved CFD under FH and CH conditions (Norouzi et al. 2016). Furthermore, the majority of the previous studies on numerical investigation of packed spheres did not validate their CFD simulations against experimental results.

The aim of this paper is to develop a robust numerical model (validated against experimental results) for permeable concrete structures using GS of different diameters (2–8 mm), under both FH and CH conditions. Monosized GS of 2, 4, 6 and 8 mm in diameter along with polysized GS combinations of 4, 6 and 8 mm in diameter (see volume ratio column in Table 1) were created using DEM simulations, with the flow through them being modelled using the resolved CFD approach under both FH and CH scenarios. GS were used in this study as data from these simple model systems can help support, validate and enhance our understanding of permeable concretes. The numerically predicted FH permeability was validated against the existing experimental results for GS in Kia et al. (2018). A general linear correlation between FH and CH permeability values for GS was established, which is not limited to the specific materials and results obtained in this study. The effect of GS diameter and mean pore size, along with the hydraulic gradient on permeability and hydraulic tortuosity was determined.

## Hydraulic Properties

### Permeability Tests and the Experimental Set-Up

**Fig. 1:**
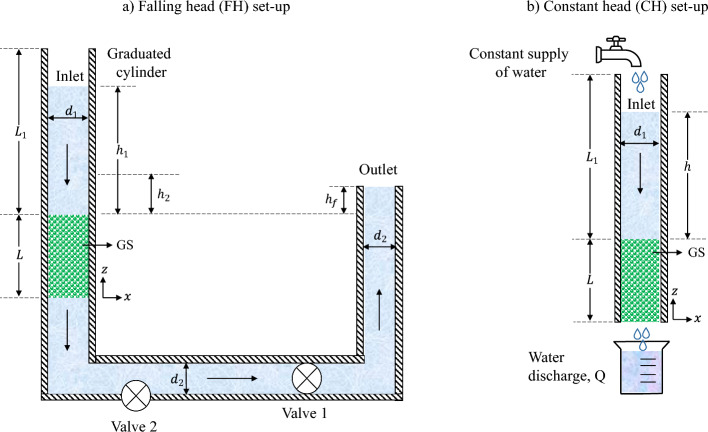
Permeability test configurations under **a**) FH; and **b**) CH conditions (Kia et al. 2018)

Permeability is a measure of the ability of a material to transmit fluids and is commonly used to characterise the flow properties of porous media. The permeability of a porous material (e.g. GS in this case) can be measured using different methods, including the FH and CH methods (see Fig. 1). In the FH method (Fig. 1a), a column of water flows through the GS, under the influence of gravity, with the water level gradually decreasing over time. The time that is required for the head to drop from an initial to a final head is recorded and used to determine permeability using Eq. 1 (Kia et al. 2018):

1 $$\begin{aligned} k_{\textrm{FH}}=\frac{A_1L}{A_2t}\ln \left( \frac{h_1}{h_2}\right) \end{aligned}$$

where $k_{\textrm{FH}}$ (m s$^{-1}$) is the permeability measured by the FH method, $A_{1}$ (m^2^) is the internal cross-sectional area of the inlet column, $A_{2}$ (m^2^) is the cross-sectional area of the GS, *L* (m) is the length of the GS, $h_{1}$ (m) is the initial head difference and $h_{2}$ (m) is the final head difference between the inlet and the bottom of the GS, and *t* (s) is the time required for the water level to drop from $h_{1}$ to $h_{2}$ (see Fig. 1a).

The experimental set-up of Kia et al. (2018), displayed in Fig. 1a, is used in this study. It consists of a GS sample cell with a length (*L*) of 150 mm, attached to a graduated cylinder at the top that is 1100 mm in length ($L_1$) and 90 mm in diameter ($d_1$). At the bottom, there is an outlet pipe with a diameter ($d_2$) of 65 mm, which is controlled by valve 1. To prevent unsaturated flow, the outlet pipe is positioned at a height ($h_f$) of 10 mm above the GS. The initial step involves preconditioning the GS by allowing water to flow through until all visible entrapped air is removed, ensuring complete saturation. To initiate the test, valve 1 is closed, and the graduated cylinder is filled with water. Valve 1 is then re-opened and the time taken for the water level to decrease from an initial head ($h_1$) of 1000 mm to a final head ($h_2$) of 250 mm is recorded. Valve 2 is used at the end of each test to drain the permeability cell. This procedure is repeated three times for each sample, and the average time (*t*) is used to determine the permeability using Eq. 1. A closely packed arrangement of GS of different diameters (2, 4 and 8 mm) is prepared by placing them in a 90$\oslash \times$ 150 mm cylinder in three equal layers, which are each compacted for 25 s using vibration. The porosity of the GS is determined from mass, volume and density measurements (Kia et al. 2018):

2 $$\begin{aligned} \epsilon = \left[ 1-\frac{m_s}{V_c\rho _s}\right] \times 100 \end{aligned}$$

where $\epsilon$ is the porosity of the GS, $V_c$ (m^3^) is the cylinder mould volume, $m_s$ (kg) is the mass of the GS and $\rho _s$ (kg m$^{-3}$) is the GS density, which equals to 2500 kg m$^{-3}$. Additional information regarding the experimental set-up can be found in (Kia et al. 2018).

In the CH method (Fig. 1b), a continuous water supply is provided at the inlet. This water supply is adjusted to maintain a constant head difference between the inlet and the bottom of the GS during the test. Once a steady flow rate is achieved, water is collected in a graduated flask for a known duration. The permeability from the CH method is then calculated by determining the total collected volume of water in the graduated flask and it is given by Eq. 3 (Bear 1988):

3 $$\begin{aligned} k_{\textrm{CH}} = \frac{Q L}{A (h+L)} \end{aligned}$$

where $k_{\textrm{CH}}$ (m s$^{-1}$) is the permeability measured by the CH method, *Q* (m^3^s$^{-1}$) is the volumetric flow rate, *L* (m) is the length of the GS, *A* (m^2^) is the cross-sectional area of the column above the GS and *h* (m) is the head difference between the inlet and the top of the GS (see Fig. 1b). The permeability $k_{\textrm{CH}}$ in m s$^{-1}$ can be converted to m^2^, by multiplying it by ${\mu }/{\rho g}$, where $\mu$ is the fluid dynamic viscosity (Pas), $\rho$ is the fluid density (kg m$^{-3}$) and *g* (m s$^{-2}$) is the acceleration due to gravity.

### Hydraulic Gradient

Hydraulic gradient (HG) represents the change in hydraulic head per unit length in the direction of flow within the GS. It is a crucial parameter as it quantifies the driving force for water flow through the GS. In the case of the FH method, it is calculated as:

4 $$\begin{aligned} \text {HG}_{\textrm{FH}}=\frac{\left[ \left( h_1+L\right) -\left( L+h_f\right) \right] -\left[ \left( h_2+L\right) -\left( L+h_f\right) \right] }{L}=\frac{h_1-h_2}{L} \end{aligned}$$

where $h_1+L$ represents the initial head difference between the inlet and the bottom of the GS in the negative *z* direction, $L+h_f$ is a constant head in the positive *z* direction at the bottom of the GS and the total initial head is equal to $h_1-h_f$. Similarly, using the same procedure, the total final head is equal to $h_2-h_f$. Therefore, the total head difference between the inlet and the bottom of the GS is equal to $h_1-h_2$.

In the case of the CH method, the HG is defined as the head difference between the inlet and the bottom of the GS divided by the length of the GS. If the bottom of the GS is open to the atmosphere, the HG would be equal to $(h+L)/L$ (see Fig. 1b). In this paper, to make an equal comparison between the FH and CH methods, the head in the positive *z* direction at the bottom of the GS in the CH method is considered to be $h_f+L$, which aligns with the FH method. Furthermore, the head difference between the inlet and the bottom of the GS in the negative *z* direction is set to be $h+L$. Therefore, the HG in the numerical model of the CH method is calculated as:

5 $$\begin{aligned} \text {HG}_{\textrm{CH}}=\frac{(h+L)-(L+h_f)}{L}=\frac{h-h_f}{L} \end{aligned}$$

According to Eq. 5, the total head at the bottom of the GS in this paper is $h-h_f$ and the permeability obtained through the CH method, as mentioned in Eq. 3, is updated as:

6 $$\begin{aligned} k_{\textrm{CH}} = \frac{Q L}{A (h-h_f)} \end{aligned}$$

### Hydraulic Tortuosity

Hydraulic tortuosity ($\tau$) is a measure of the degree of tortuosity in the hydraulic pathways of GS. It is defined as the proportion of space-averaged velocity magnitude to the velocity in the principle direction of fluid flow (Duda et al. 2011).

7 $$\begin{aligned} \tau =\frac{\langle v \rangle }{\langle v_z \rangle } \end{aligned}$$

where $\langle v \rangle$ (m s$^{-1}$) is the space-averaged velocity within the GS and $\langle v_z \rangle$ (m s$^{-1}$) is the space-averaged velocity in the main flow direction (i.e. along the *z* coordinate). The space average represents the average value of velocity over a specified volume of GS, denoted as *V*. The space average is given as:

8 $$\begin{aligned} \langle v \rangle =\frac{1}{V}\int v {\textrm{d}}V \end{aligned}$$

It should be noted that in the CFD simulations, *dV* is located in the pore space between the GS and is equivalent to the volume of an individual cell.

The validity of Eq. 7 relies on two assumptions of: (i) fluid is incompressible; and (ii) re-entrant flow is absent (Duda et al. 2011). Re-entrant flow typically occurs in fluids where rapid shifts in pore size or the presence of blind pore spaces^1^ can induce the formation of re-circulation zones, often referred to as eddies. However, for flow with low Reynolds numbers, especially in flows through GS, the regions where re-entrant flow occurs constitute only a small fraction of the overall GS volume. Consequently, Eq.  (7) remains a valuable tool for approximating hydraulic tortuosity in situations characterised by low modified Reynolds numbers (Re$'$ ranging from 1 to 10). In this context, the modified Reynolds number for GS is defined as (Kia et al. 2018):

9 $$\begin{aligned} {\text {Re}}'=\frac{\rho u_s D}{(1-\epsilon )\mu } \end{aligned}$$

where $\rho$ (kg m$^{-3}$) is density of the fluid, $u_s$ (m s$^{-1}$) is the superficial velocity, *D* (m) is the diameter of GS, $\epsilon$ is the porosity of the GS and $\mu$ (Pa s) is the dynamic viscosity of the fluid. It is worth noting that the superficial velocity is the average velocity of water, assuming it flows uniformly through the entire cross-sectional area of the GS, without considering the intricate flow paths within the pores.

## Numerical Simulations

### Governing Equations

Three-dimensional resolved numerical simulations of the flow through the GS are performed using an open-source CFD software, OpenFOAM 5.0 (Weller et al. 1998), on the high-performance computing system of Imperial College London. As the CH method concerns a constant water level, this approach can be modelled using a steady-state, single-phase solver with the whole fluid domain consisting of water. In contrast, as the FH method involves a falling water level, it requires a transient, two-phase solver and treatment of the interface between water and air. The governing equations for the CH and FH methods are outlined below and all simulations reported in the present study employed the resolved CFD approach.

#### CH Method

The fluid motion for the CH simulations assumes an incompressible steady-state flow and is governed by the Navier–Stokes equations:

10 $$\begin{aligned} \nabla \cdot \textbf{U}=0 \end{aligned}$$

11 $$\begin{aligned} (\textbf{U} \cdot \nabla ) \textbf{U}= -\frac{1}{\rho }\nabla p + \nu \nabla ^2 \textbf{U}+ \textbf{g} \end{aligned}$$

where $\textbf{U}$ (m s$^{-1}$) is the velocity vector, *p* ( Pa) is the pressure, $\nu$ (m^2^s$^{-1}$) is the kinematic viscosity, $\textbf{g}$ (m s$^{-2}$) is the gravitational acceleration vector and $\nabla$ is the vector differential operator. Equation 10 is the continuity equation, which ensures that mass is conserved for an incompressible flow. Equation 11 is the momentum equation, which describes how the velocity changes over time due to the pressure and viscous forces (Moukalled et al. 2016). Equations 10 and 11 hold true for steady-state conditions when the CH method is required, therefore the single-phase steady-state solver named buoyantBoussinesqSimpleFoam is used.

#### FH Method

For transient conditions or the FH approach, it is necessary to model the air and water phases separately. For the FH simulations in this study, the multi-phase transient interFoam solver, with the volume of fluid method, is utilised to accurately track and analyse the interface between the water and air phases. In this method, the interface between the fluids is tracked through every cell in the computational grid. In order to track the interface, a volume fraction conservation equation is solved for each phase, alongside a single set of momentum equation with additional source terms to account for fluid surface tension and wall adhesion.

12 $$\begin{aligned} \nabla \cdot \textbf{U}=0 \end{aligned}$$

13 $$\begin{aligned} \frac{\partial (\rho \textbf{U})}{\partial t}+ \nabla \cdot (\rho \textbf{U} \textbf{U}^T)= -\nabla p + \nabla \cdot (\mu ( \nabla \textbf{U}+ \nabla \textbf{U}^T)) + \rho \textbf{g}+ f_{\sigma } \end{aligned}$$

Equation 12 is the constant-density continuity equation and Eq. 13 is the momentum equation for two fluids, where $\mu$ (Pa.s) is the dynamic viscosity of the fluids, $f_{\sigma }$ (Nm$^{-3}$) is the surface tension between the water and air phases and superscript *T* represents the transpose operator. The density in this equation is defined as:

14 $$\begin{aligned} \rho =\alpha \rho _1 + (1-\alpha )\rho _2 \end{aligned}$$

where $\rho _1$ (kg m$^{-3}$) and $\rho _2$ (kg m$^{-3}$) are the density of water and air, respectively. The volume fraction $\alpha$ represents the portion of each phase in a given computational cell, ranging from 0 to 1, where $\alpha = 0$ indicates a cell completely filled with air and $\alpha = 1$ represents a cell entirely filled with water. The surface tension in Eq. 13 is modelled as a continuum surface force. Further information on the surface tension is provided in Brackbill et al. (1992). An additional equation for $\alpha$ needs to be solved to determine the location of the interface between the two fluids.

15 $$\begin{aligned} \frac{\partial \alpha }{\partial t}+\nabla \cdot ( \alpha \textbf{U})=0 \end{aligned}$$

This equation can be interpreted as the conservation of the mixture components along the trajectory of a fluid parcel.

### Glass Sphere Generation

For GS generation, DEM was applied and the STAR-CCM+ software from Siemens PLM was used (Siemens Digital Industries Software 2022). DEM is a soft-body approach pioneered by Cundall and Strack (1979), which considers spheres as deformable bodies. The interactions between these spheres are calculated using Newton’s second law.

16 $$\begin{aligned} m_i \frac{{\textrm{d}}\textbf{u}_i}{{\textrm{d}}t}= \textbf{F}_{s}+\textbf{F}_b \end{aligned}$$

where $m_i$ (kg) is the mass of the *i*th sphere, $\textbf{u}_i$ (m s$^{-1}$) is the *i*th sphere velocity, $\textbf{F}_{s}$ (N) represents the surface forces and $\textbf{F}_{b}$ (N) consists of gravity and contact forces.

**Fig. 2:**
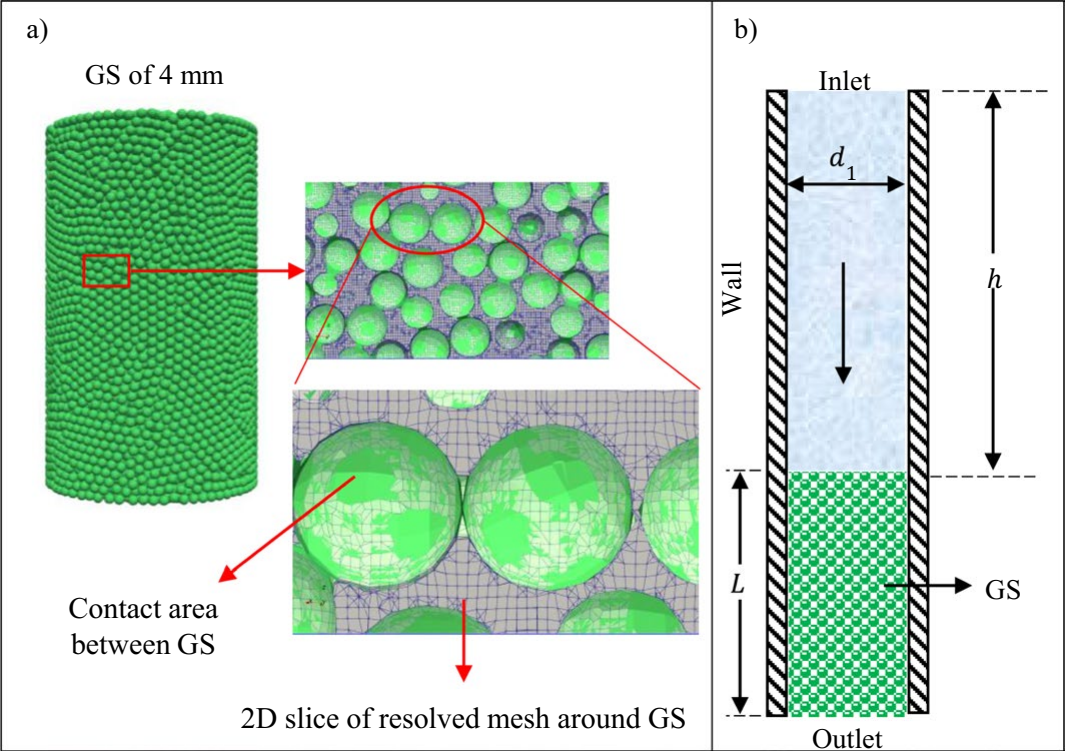
Monosized GS of 4 mm diameter, with **a**) the corresponding resolved mesh in a vertical cross section; and **b**) the CFD simulation domain schematic

In STAR-CCM+, the initial step involves creating a cylinder to serve as the container for the GS. The cylinder has dimensions 90$\oslash \times$ 200 mm and is initially empty, as no fluid simulation is performed at this stage. The walls of the cylinder are rigid enough, with a shear modulus of $10^{+13}$ Pa, to prevent the GS from extruding through. The GS are generated from a series of randomly distributed points located at the top of the cylinder at a frequency of 1000 per second. These GS have a settling velocity in the *z*-direction of − 1 m s$^{-1}$, chosen such that they gradually form a compact packed bed. The density, Poisson’s ratio and shear modulus of GS are considered to be 2500  kg m$^{-3}$, 0.245 and 10 GPa, respectively. The resulting monosized GS of 4 mm diameter are depicted in Fig. 2a. It should be noted that the GS sample length in this figure is 150 mm.

### Computational Domain and Boundary Conditions

The schematic of the computational domain is shown in Fig. 2b. The geometry is generated in two steps. Firstly, an unstructured GS is created using Star-CCM+ (Flaischlen and Wehinger 2019), as described in Section 3.2. Subsequently, the generated GS is imported into OpenFOAM and the snappyHexMesh utility is employed to subtract the GS from the background mesh (see Fig. 2a). As a result, the domain exclusively consists of fluid cells. Furthermore, as can be seen in Fig. 2a, the flow on the surface of each GS is resolved to ensure accurate simulations.

In both the CH and FH scenarios, a Neumann boundary condition is applied to the inlet and outlet velocities, with the gradient value set to zero. This allows for the calculation of the velocity, based on the pressure difference between the inlet and the outlet. For both cases, the total inlet pressure is set to zero, while the outlet pressure is adjusted to 1600 Pa to account for the hydrostatic pressure at the outlet, as shown in Fig. 1a ($h_f+L = 0.16$ m). The no-slip velocity boundary condition is applied to the surface of the GS, meaning that the velocity of the fluid at these boundaries is set to zero. Furthermore, a zero gradient condition is applied to the pressure on the walls and the cells adjacent to the GS surface, ensuring that the normal pressure gradient at these boundaries remains zero. To ensure numerical stability during transient simulations (i.e. for the FH method, see Sect. 3.1.2), the Courant Number and simulation time step ($\Delta t$) are assessed. For example, in the case of a monosized GS with 8 mm diameter, the mean and maximum Courant numbers are 0.00131 and 0.99810, respectively, whilst the mean and maximum interface Courant numbers are 0.00016 and 0.71950, respectively, with $\Delta t$ set to 8.56 $\times 10^{-6}$ s (Moukalled et al. 2016).

### Pore Size Distribution

Pore size distribution provides information about the distribution and frequency of pores of varying sizes within the GS, which is crucial for understanding its permeability and hydraulic tortuosity (Neithalath et al. 2010b). To determine the pore size distribution, GS described in Sect. 3.2 are imported into the Avizo software (Avizo Software 2019). Within Avizo, a three-dimensional representation of the GS is voxelized, with each voxel being designated as either a pore (assigned a value of 1) or a part of the GS matrix (assigned a value of 0). By selectively focusing on the voxels attributed to the pore component, data related to the GS pore volume and pore size distribution are extracted. Furthermore, to mitigate potential edge effects, a core subsample from the packed bed is utilised. This subsample consists of a 85 mm diameter cylinder, which is slightly smaller than the overall cylinder diameter of 90 mm. In addition, to provide a more representative pore diameter for GS of different size distributions, the arithmetic mean value of the pore sizes is chosen in this study (see Table 1). The pore size standard deviation (SD) in Table 1 represents the spread of the GS particle sizes.

## Results and Discussion

The hydraulic performance of the GS was investigated through a combination of experimental and numerical analyses. The experimental measurements of Kia et al. (2018) determined the FH permeability of the GS with diameters of 2, 4 and 8 mm. The numerical simulations were performed under FH and/or CH permeability test conditions, for mono and polysized GS of different diameter, porosity, mean pore size and volume ratio, as outlined in Table 1. Cases 1–4 in Table 1 are monosized GS of 2, 4, 6 and 8 mm in diameter, while cases 5–11 are polysized GS of 4, 6 and 8 mm in diameter with size distributions indicated as a percentage in the order of 4 mm:6 mm:8 mm. For example, a size distribution of 10:20:70 indicates that 10$\%$ of the volume consists of 4 mm diameter GS, 20$\%$ consists of 6 mm diameter GS, and 70$\%$ consists of 8 mm diameter GS. The existing experimental results were used to validate the developed numerical model.

**Table 1:** Monosized and polysized GS characteristics and size distributions used in the numerical simulations

Case	Volume ratio (%)	Porosity (%)	Pore size mean ± SD (mm)	Number of GS (2 mm)	Number of GS (4 mm)	Number of GS (6 mm)	Number of GS (8 mm)
1	100$\%$ 8 mm	35.20	3.30 ± 2.10	–	–	–	2314
2	100$\%$ 6 mm	34.00	2.60 ± 2.03	–	–	5585	–
3	100$\%$ 4 mm	33.75	1.70 ± 1.67	–	18,920	–	–
4	100$\%$ 2 mm	33.00	1.05 ± 1.02	153,071	–	–	–
5	10:20:70	33.00	2.80 ± 2.59	–	1982	1188	1643
6	20:10:70	32.20	2.52 ± 2.35	–	3969	572	1683
7	10:70:20	33.00	2.13 ± 1.71	–	1924	4047	444
8	20:70:10	32.50	1.86 ± 1.80	–	3908	4035	219
9	70:10:20	32.70	1.66 ± 1.38	–	13,773	526	459
10	70:20:10	32.80	1.61 ± 1.56	–	13,659	1094	230
11	33:33:34	32.50	1.81 ± 1.68	–	6578	1940	769

The mean pore size and standard deviation (SD) were calculated using the Avizo software (see Sect. 3.4) and the number of GS is an output from the DEM simulations (see Sect. 3.2)

### Experimental Permeability

**Fig. 3:**
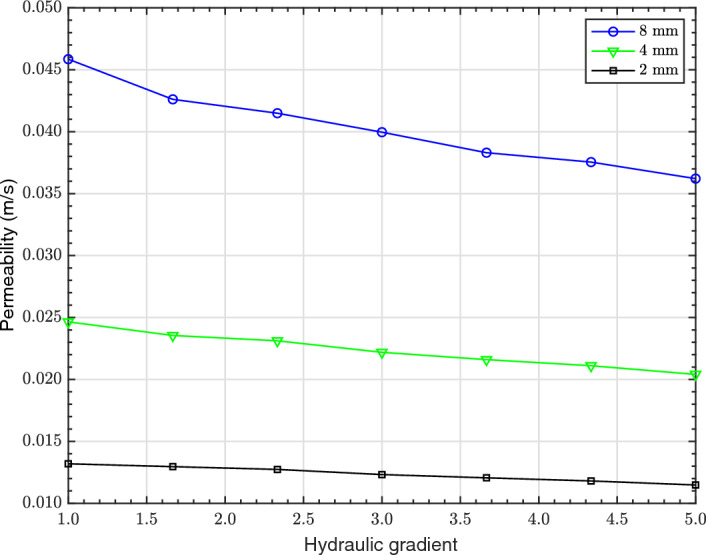
Correlation between experimental FH permeability and HG for GS of 2, 4 and 8 mm in diameter (Kia et al. 2018)

Figure 3 presents the experimental FH permeability against different HG for GS of 2, 4 and 8 mm in diameter, obtained from Kia et al. (2018) (who also made comparisons to the Kozeny–Carman equation, which are not repeated herein). The porosity of the 2, 4 and 8 mm monosized GS are 33.36$\%$, 33.83$\%$ and 35.74$\%$, respectively (Kia et al. 2018). As expected, the permeability of the larger diameter GS is higher than that of the smaller diameter, attributed to the greater mean pore size between the larger diameter GS (see Table 1), allowing water to more easily flow through the packed bed. Although the experimental measurements provide valuable information about the permeability, they do not provide insight into the hydraulic tortuosity of GS, for which CFD modelling results will be relied upon.

### Porosity of the Packed Glass Spheres

The porosity of the GS for different size distributions within the numerical simulations is presented in Table 1 and was calculated as the ratio of the pore volume to the total volume (where the pore volume was found by subtracting the volume of all glass spheres from the total volume). As can be observed from Table 1 and Sect. 4.1, the porosity of the monosized GS with 8 mm diameter matches the value obtained in the experiment (Kia et al. 2018). However, the porosity of the monosized GS with diameters of 2 and 4 mm, within the numerical simulations, slightly exceeds the experimental measurements by 2.1$\%$ and 2.5$\%$, respectively (Kia et al. 2018). In the experiments of Kia et al. (2018), various replicate samples were examined and they exhibited a 2.5% variability in the porosity. Given that the numerical discrepancy falls within this 2.5$\%$ experimental variability, the validity of the numerically generated GS is maintained. Furthermore, it can be observed from Table 1 that the porosity of the polysized GS is slightly lower than that of the monosized GS. The porosity of the polysized GS cannot be decreased below 32$\%$, which is a limitation for modelling low porosity permeable concretes. However, given permeable concretes have porosities in the range of 15–35$\%$ (Kia et al. 2021a, b, 2022), the current GS configurations are applicable to the higher end of this porosity range.

### CFD Verification Through Grid Convergence Test

A series of grid convergence tests were conducted, using the CH approach, for different monosized (2, 4, 6 and 8 mm in diameter) and polysized (4, 6 and 8 mm in diameter) GS combinations to ensure the reliability of the CFD results. The number of cells were progressively refined (see Table 2), with the permeability values monitored until further refinement had no effect on the permeability values. The suitable number of cells for each GS diameter is denoted in bold in Table 2 for ease of identification. For example, for monosized GS of 8 mm in diameter, a mesh with approximately 5.9 million cells was found to be sufficient with a relative error of 1.75$\%$. It is important to highlight that in all cases the relative error was less than 2.75$\%$.

**Table 2:** Grid convergence test for CH CFD of monosized and polysized GS combinations

Case	Diameter of GS	Number of cells	Permeability		Relative error
	(mm)	(million)	(m s$^{-1}$)	($\times$10^-9^ m^2^)	(%)
1	8	1.44	0.0181	1.85	33.00
		3.10	0.0250	2.55	7.44
		**5**.**90**	**0**.**0266**	**2**.**71**	**1**.**75**
		9.80	0.0271	2.76	–
2	6	4.90	0.0180	1.83	18.02
		8.40	0.0206	2.10	6.05
		**13**.**30**	**0**.**0213**	**2**.**17**	**2**.**59**
		17.50	0.0219	2.23	–
3	4	13.80	0.0127	1.29	18.22
		18.90	0.0145	1.48	6.55
		**24**.**80**	**0**.**0152**	**1**.**55**	**2**.**47**
		31.60	0.0156	1.59	–
4	2	56.50	0.0044	0.45	37.00
		87.00	0.0062	0.63	11.50
		105.00	0.0067	0.68	3.50
		**124**.**00**	**0**.**0070**	**0**.**71**	–
5	Polysized volume ratio 10:20:70	17.20	0.0225	2.29	4.14
		**19.20**	**0**.**0231**	**2**.**35**	**1**.**56**
		22.90	0.0233	2.38	0.40
		28.30	0.0234	2.39	–
6	Polysized volume ratio 20:10:70	16.90	0.0207	2.11	4.70
		**19**.**40**	**0**.**0213**	**2**.**17**	**1**.**90**
		23.50	0.0216	2.20	0.53
		29.00	0.0217	2.21	–
7	Polysized volume ratio 10:70:20	17.10	0.0205	2.09	4.67
		**20.00**	**0**.**0210**	**2**.**14**	**2**.**04**
		24.30	0.0214	2.18	0.51
		30.00	0.0215	2.19	–
8	Polysized volume ratio 20:70:10	16.70	0.0189	1.93	5.76
		**20**.**20**	**0**.**0195**	**1**.**99**	**2.70**
		24.80	0.0199	2.03	0.81
		30.10	0.0201	2.05	–
9	Polysized volume ratio 70:10:20	15.10	0.0155	1.58	10.65
		20.80	0.0166	1.69	4.73
		**26**.**60**	**0**.**0171**	**1**.**74**	**1**.**70**
		33.50	0.0174	1.77	–
10	Polysized volume ratio 70:20:10	15.10	0.0155	1.58	10.42
		20.80	0.0165	1.68	4.87
		**26**.**70**	**0**.**0170**	**1**.**73**	**1**.**75**
		33.70	0.0173	1.76	–
11	Polysized volume ratio 33:33:34	16.70	0.0186	1.90	6.33
		**20**.**30**	**0**.**0193**	**1**.**97**	**2**.**75**
		25.20	0.0197	2.01	0.75
		31.40	0.0199	2.03	–

### CFD Validation by Comparison with Experimental Results

**Fig. 4:**
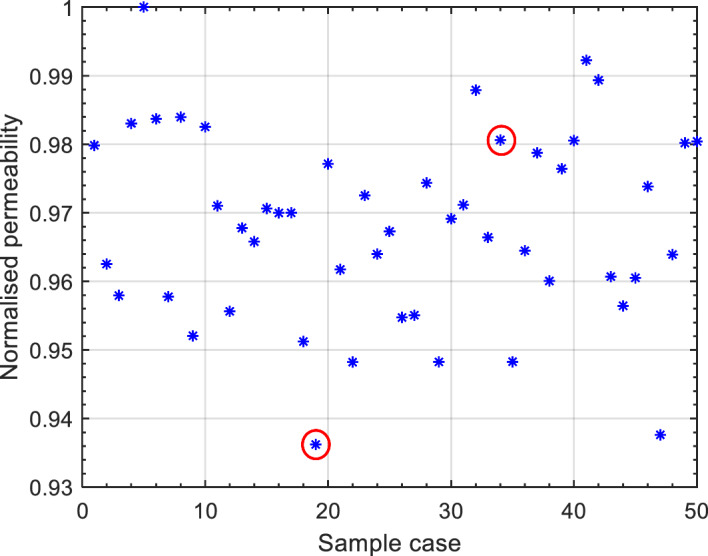
Normalised permeability (sample permeability divided by the maximum permeability) obtained from the CH numerical simulations for 50 different randomly packed monosized GS of 8 mm in diameter (35.2$\%$ porosity)

The permeability values obtained through CFD simulations were compared against the existing experimental data (Kia et al. 2018) in order to validate the numerical model results. To perform this validation, it was first necessary to examine the variability introduced by the random packing process within the unstructured GS. This is an essential step as in the experimental measurements a single randomly packed sample was investigated. The permeability of 50 different randomly packed monosized GS of 8 mm in diameter was simulated under CH conditions (see Fig. 4). As it can be seen from Fig. 4, while the number and diameter of the randomly generated GS samples remain consistent, there is a maximum difference of 6.5$\%$ in the permeability values calculated from the CH method. Based on this analysis, samples 19 and 34 (circled in red in Fig. 4) were randomly selected for the validation process. To assess the accuracy of the CFD method, the permeability values obtained from the experimental FH measurements (Kia et al. 2018) were compared against the numerical results for the 8 mm diameter monosized GS.

**Fig. 5:**
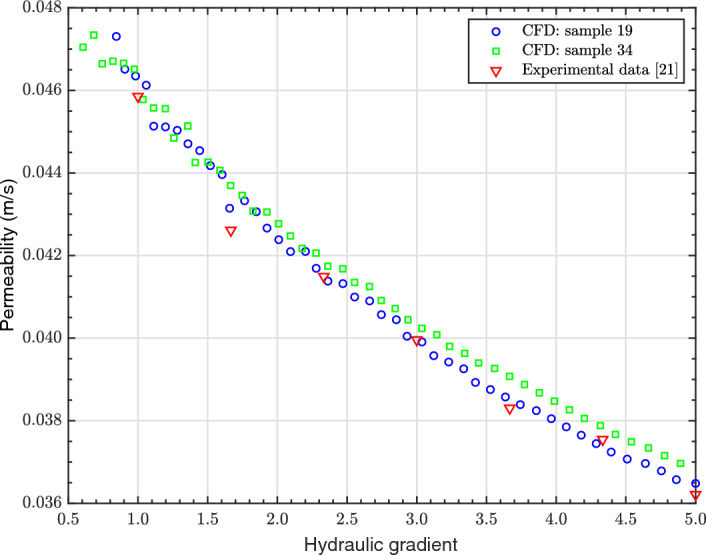
FH permeability validation between the CFD simulations and the experimental measurements of Kia et al. (2018) for monosized GS of 8 mm in diameter

The validation results, depicted in Fig. 5, demonstrate that the error between the experimental and numerical permeability values is less than 4$\%$. This finding provides confirmation of the reliability and validity of the CFD simulations. It is important to emphasise that the resolved CFD model validation was achieved solely by creating a geometry and setting the applied pressure across the GS, without conducting any calibrations, empirical corrections or other adjustments to the model.

### Permeability of the Packed Glass Spheres

The numerical permeability of GS was investigated under the CH conditions as opposed to the FH due to the significant difference in computational run times between the two types of simulations, with the FH method requiring more computational resources. For example, the CH numerical simulation time for monosized GS of 8 mm in diameter was approximately 1.5 h, using 64 cores and 64 GB RAM. In contrast, the equivalent FH simluation took approximately 370 h, despite using 252 cores and 100 GB RAM. Furthermore, as the permeability values of GS with different size distributions were compared amongst each other, the choice of the test method (e.g. FH or CH) would not have a significant impact.

**Fig. 6:**
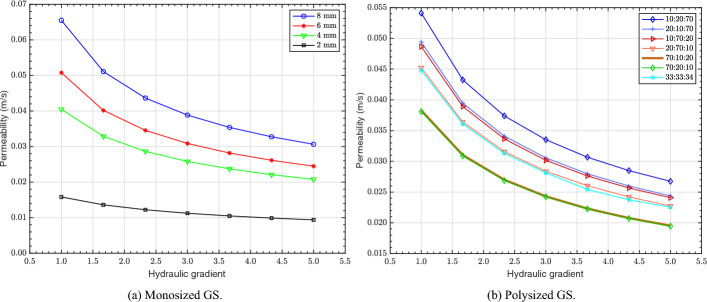
Permeability of GS under different HGs using CH CFD simulations

The permeability of the monosized GS of different diameters (2, 4, 6 and 8 mm) under different HGs using CH CFD simulations is displayed in Fig. 6a. As expected, permeability increased with increase in the GS diameter, in line with the experimental FH permeability trend (see Fig. 3). This is due to the higher mean pore size of the GS with larger diameters, 3.30 mm versus 1.05 mm for 8 and 2 mm diameter GS, respectively (see Table 1), leading to an increase in the subsequent permeability values. Permeability is therefore directly proportional to the mean pore diameter for monosized GS.

Figure 6b illustrates the permeability of the polysized GS under different HGs using the CH method. It can be seen that the polysized combinations with a higher proportion of 8 mm diameter GS, such as the sample with 10:20:70 volume ratio, exhibit the highest permeability attributed to their larger mean pore size (see Table 1). As the contribution of 4 mm GS in the polysized combinations increases, the permeability values decrease due to the reduction in the mean pore size. Furthermore, when the majority of a polysized combination consists of 4 mm GS, any variations in the volume ratio of the 6 and 8 mm diameter GS does not have a considerable impact on the permeability values. This is due to the 4 mm diameter GS filling most of the open voids and determining the flow rate. In addition, the permeability values of the samples with 33:33:34 and 20:70:10 volume ratio are almost identical due to their mean pore sizes being very similar (see Table 1).

The permeability values of both monosized and polysized GS are influenced by the HG. As it can be seen from Figs. 6a and  6b, the permeability values decrease with increase in the HG. Permeability represents the ease in which fluid flows through a porous material and this is governed by factors such as pore size, connectivity and the nature of the fluid flow. At low HGs, the flow predominantly remains linear and follows Darcy’s law, maintaining a stable permeability. However, an increase in the HG, leads to an increase in velocity, leading to the inertial and turbulent effects becoming more prominent. This leads to the formation of eddies, vortices and flow separations within the pore spaces, increasing energy dissipation, where kinetic energy is lost due to turbulence and friction. As a result, the fluid faces greater resistance, requiring more energy to sustain the flow, which effectively reduces the permeability.

**Fig. 7:**
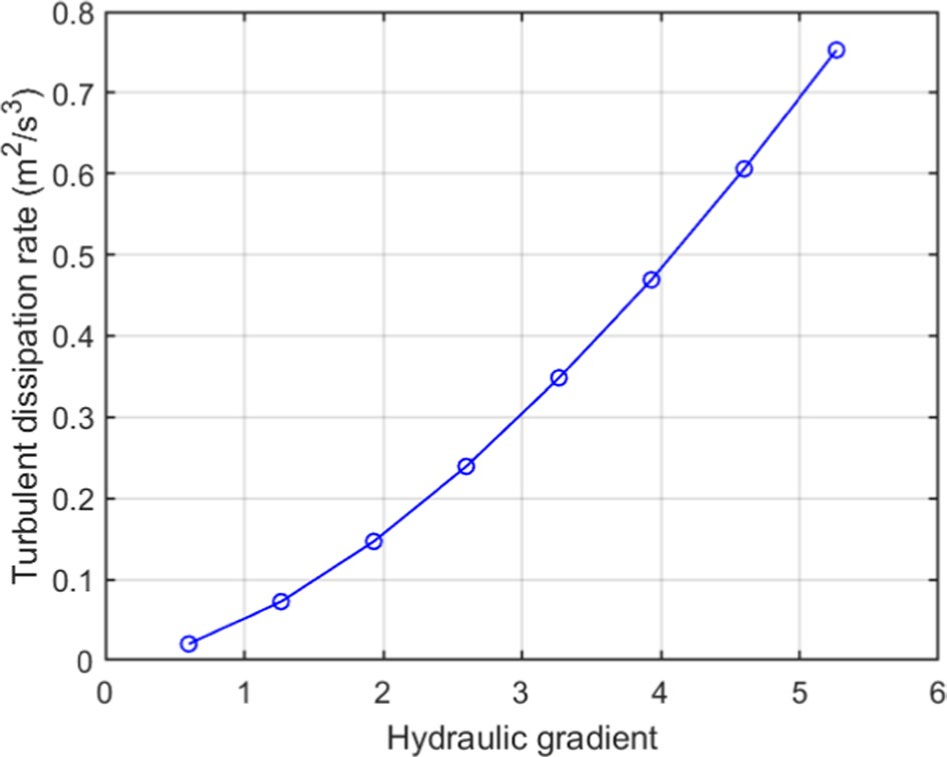
Turbulent dissipation rate variation with hydraulic gradients for monosized GS of 8 mm

To quantify this, the turbulent dissipation rate was calculated for monosized packed GS of 8 mm by using Eq. 17.

17 $$\begin{aligned} \epsilon = \frac{C_\mu ^{3/4}\cdot k^{3/2}}{L} \end{aligned}$$

where $C_\mu$ is an empirical turbulence model constant and is equal to 0.09, *k* is the turbulent kinetic energy (m^2^s$^{-1}$) and *L* (m) is the turbulent length scale, which is equal to the mean pore size value of the packed GS (see Table 1). Figure 7 shows the variation in the turbulent energy dissipation with the HG and demonstrates that increasing the HG leads to an increase in the turbulent dissipation rate.

### FH and CH Permeability Correlation

**Fig. 8:**
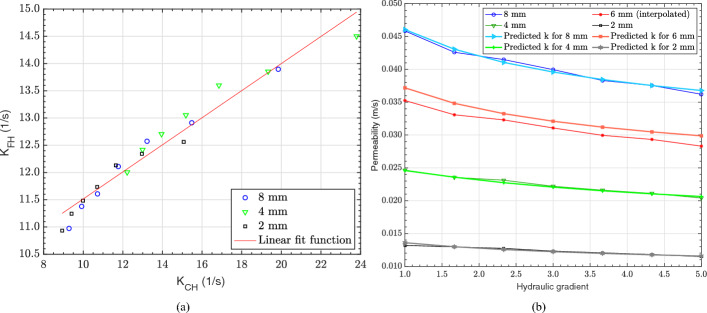
**a**) Correlation between the experimental FH (Kia et al. 2018) and numerical CH permeability divided by the mean pore size (denoted as K) for monosized GS; **b**) Comparison between the experimental FH permeability and that predicted by the linear correlation function in Eq.  (18)

Permeability was found to be directly proportional to the mean pore size (see Sect. 4.5) and it was therefore decided that this parameter should be included as part of establishing the correlation between the FH and CH permeability values. The CH permeability determined from the CFD simulations (see Fig.  6a) and the FH permeability obtained from the experimental data of Kia et al. (2018) (see Fig.  3) were divided by the mean pore size of the GS (see Table 1). Figure 8a, shows a strong linear correlation between the experimental FH and numerical CH permeability values divided by the mean pore size of monosized GS at different HGs. This linear correlation function is given by

18 $$\begin{aligned} {K}_{\textrm{FH}}= 0.25~{K}_{\textrm{CH}}+9.03 \end{aligned}$$

where ${K}_{\textrm{FH}}$ and ${K}_{\textrm{CH}}$ represent the permeability values obtained from the FH and CH methods divided by the mean pore size of the corresponding monosized GS from Table 1, respectively. To validate the accuracy of this function, the CH permeability values obtained from CFD simulations were used to predict the FH permeability values. These predicted values were then compared against the experimental FH data (Kia et al. 2018) for GS of 2, 4, 6 and 8 mm in diameter. It is worth noting that as there are no experimental FH permeability values for the 6 mm diameter GS, this data was obtained through linear interpolation between the experimental FH permeability values for GS of 2, 4 and 8 mm in diameter. As demonstrated in Fig. 8b, the predicted FH permeability values obtained from the correlation function in Eq. (18) are closely aligned both with the experimental and the interpolated permeability values. As such the root mean squared error between the experimental and the predicted permeability values for GS of 2, 4, 6 and 8 mm in diameter are 1.39$\%$, 0.82$\%$, 4.49$\%$ and 0.92$\%$, respectively, when normalised by the experimental measurements.

It is important to note the range of validity for Eq. 18 and compare it against similar expressions within the literature. Equation 18 applies to flows through permeable concretes with HGs ranging between 1 and 5, mean pore sizes ranging from 1.05 to 3.3 mm, and porosity values between 33 and 35.2%. The range of porosity may appear small, however, it is important to note that this is typical for permeable concretes, which are the focus of the present study. Furthermore, the offset value of 9.03 may appear large, however, this is due to the normalisation by the mean pore size within Eq. 18. The absolute offset value is approximately 0.02, which affirms the physical expectation that permeability values from FH and CH methods both tend to zero simultaneously. Equation 18 differs from previously published correlations (Zhang et al. 2020; Akkaya and Çağatay 2021; Sandoval et al. 2017) primarily because prior studies assumed a fixed HG while deriving their relationships between CH and FH permeability methods. In contrast, the present study considers a range of HGs, incorporates samples with different porosities and further normalises permeability values using mean pore size. This approach provides a broader and more generalised correlation. Zhang et al. (2020) and Akkaya and Çağatay (2021) established a linear relationship between the absolute permeabilities $k_{\textrm{FH}}$ and $k_{\textrm{CH}}$. However, their equations exhibited a steeper slope, indicating a stronger sensitivity of $k_{\textrm{FH}}$ to changes in $k_{\textrm{CH}}$. In contrast, our correlation shows a more moderate slope, likely due to the normalisation of permeability with the mean pore size. The proposed relationship by Sandoval et al. (2017) suggests that $k_{\textrm{CH}}$ increases at a nonlinear rate with $k_{\textrm{FH}}$, which contradicts the trends observed in Zhang et al. (2020), Qin et al. (2015), Batezini and Balbo (2015), and Akkaya and Çağatay (2021). Sandoval et al. (2017) also reported that the permeability values obtained using the FH method were significantly lower than those obtained using the CH method. However, no explanation was provided for this discrepancy. It is therefore believed that the work by Sandoval et al. (2017) is only applicable to the specific experimental set-up used in that study.

Thus far, this section has considered the idealised case of GS, however, it is also of interest to consider whether the relationship between CH and FH is applicable to real permeable concretes. Considering the experiments of Kia et al. (2018), the FH permeability of a permeable concrete sample with a porosity of 32% is 0.016 m s$^{-1}$. The 70:20:10 volume ratio polysized GS combination has the lowest mean pore size of 1.61 mm, indicating that its pore structure is tortuous, similar to permeable concretes (see Sect. 3), and has a porosity value of 32.8% that is close to the permeable concrete sample. The numerical CH permeability of the 70:20:10 volume ratio polysized GS is 0.017 m s$^{-1}$, which using Eq. (18), corresponds to a FH permeability of 0.019 m s$^{-1}$ that is very close to the FH permeability of the 32% porosity permeable concrete sample. This indicates that the developed numerical model in this study can be used for permeable concretes of higher porosity with future studies exploring alternative shapes or packing configurations to enhance the applicability of the model to all permeable concrete structures.

**Table 3:** Hydraulic tortuosity of monosized and polysized GS combinations in the CH scenario

Case	Volume ratio (%)	Hydraulic tortuosity
1	100$\%$ 8 mm	1.2197
2	100$\%$ 6 mm	1.2273
3	100$\%$ 4 mm	1.2369
4	100$\%$ 2 mm	1.2831
5	10:20:70	1.2256
6	20:10:70	1.2338
7	10:70:20	1.2334
8	20:70:10	1.2360
9	70:10:20	1.2391
10	70:20:10	1.2405
11	33:33:34	1.2340

### Tortuosity of the Packed Glass Spheres

Hydraulic tortuosity is a key parameter in porous media analyses, as it characterises the complexity of the flow paths within the pore structure. In this study, hydraulic tortuosity was determined using a subsample to minimise the boundary effects and was evaluated under a controlled hydraulic gradient and modified Reynolds number to ensure consistency in the flow conditions.

**Fig. 9:**
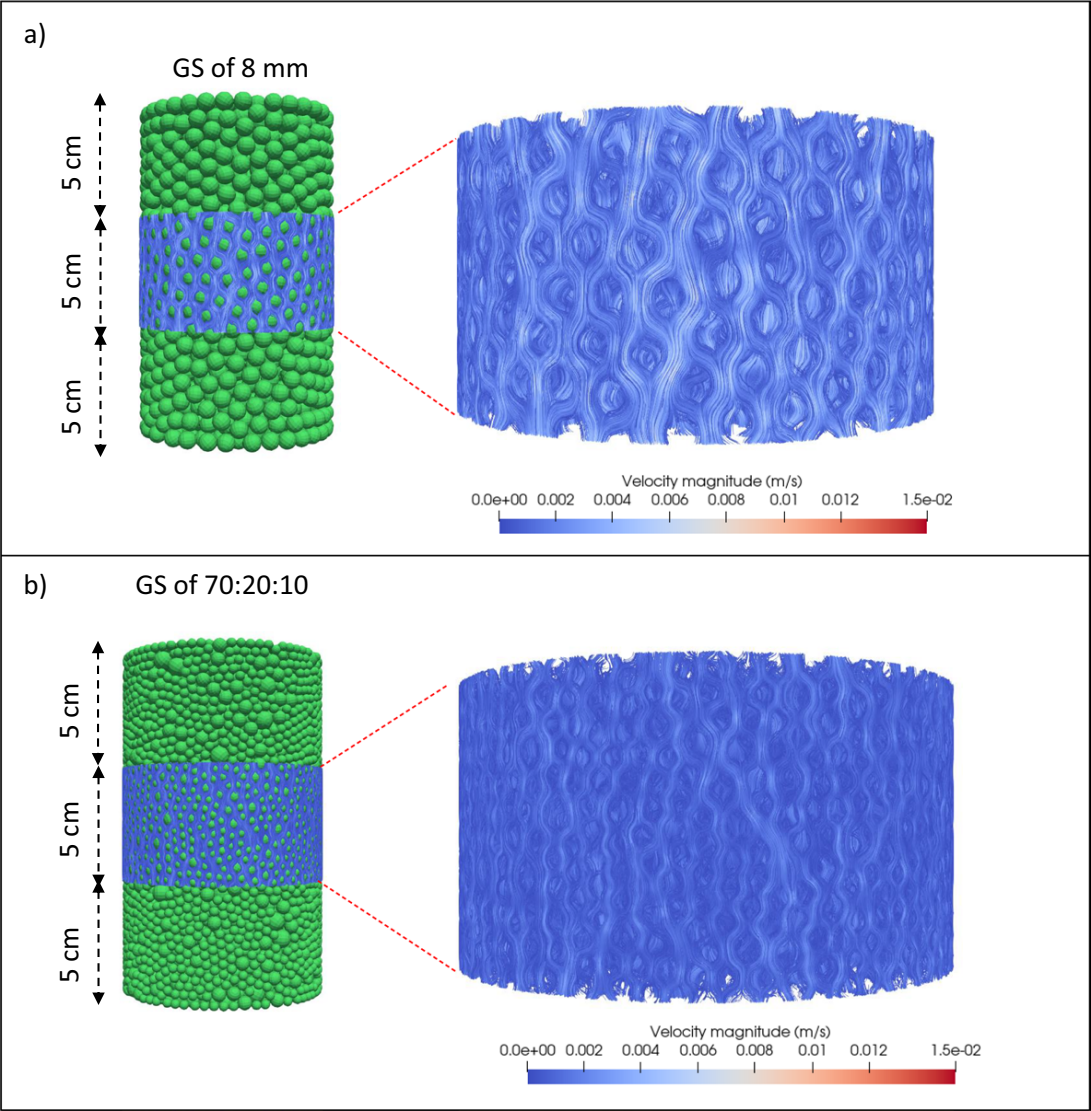
Flow streamlines obtained from CH CFD simulations within a subsample of **a**) monosized 8 mm diameter GS; and **b**) polysized (70:20:10 volume ratio) GS combination

The results in Table 3 indicate a clear inverse relationship between the hydraulic tortuosity and the GS diameter for all monosized GS. As the GS diameter increases, the flow paths become more direct, reducing the deviation from a straight path and leading to lower tortuosity values. This is primarily due to the larger pore spaces present between larger GS, allowing the water to travel through the medium with fewer directional changes. For polysized combinations, an increase in the hydraulic tortuosity values is observed with increase in the smallest GS of 4 mm in diameter. This is due to the smaller GS occupying interstitial voids between the larger GS, thereby disrupting the otherwise direct flow paths and introducing additional convolutions in the streamlines. The increase in hydraulic tortuosity is particularly significant in cases where the 4 mm GS proportion is the highest, as these particles create more obstructions and force the fluid to navigate through a more intricate pore network. Figure 9 presents a more detailed visualisation of the flow streamlines for both monosized GS of 8 mm in diameter and a polysized GS combination with a 70:10:20 volume ratio. When comparing the monosized 8 mm diameter GS with the 70:10:20 volume ratio polysized GS combination, it is evident that the streamlines in the 8 mm diameter GS follow a more direct path with minimal curvature. Conversely, in the GS combination, the streamlines exhibit a more winding trajectory, confirming the increased hydraulic tortuosity. From a practical perspective, these findings have important implications for the design of permeable concretes and other engineered porous media. If higher tortuosity is desired (e.g. for enhanced filtration efficiency or increased residence time in reactive porous systems), incorporating a higher proportion of smaller particles within the packing structure can achieve this. Conversely, for applications such as permeable concretes where lower hydraulic resistance and higher permeability values are required, a monosized or sparsely packed structure with larger particles is preferable.

## Conclusion

A robust numerical model, validated against experimental results, was developed for permeable concrete structures using GS of different diameters. Monosized and polysized GS were used in this study to represent an idealised permeable concrete structure and the effect of the pore characteristics, including porosity and mean pore size, as well as the hydraulic gradient on permeability and hydraulic tortuosity was determined. DEM was used to generate the GS, whilst CFD was employed to simulate the water flow through the packed bed under both FH and CH conditions. The CFD-simulated permeability values matched very closely to that of the experimental data, without necessitating any adjustments or calibrations. Subsequently, this validated resolved CFD model was leveraged to investigate the effect of different HGs on GS’ permeability and hydraulic tortuosity. The permeability of both monosized and polysized GS was found to decrease with increase in the HG. However, the permeability was found to increase with increase in porosity and mean pore diameter. An inverse relationship was observed between permeability and the HG as well as between tortuosity and GS diameter. Furthermore, a higher proportion of smaller diameter GS in polysized combinations led to a decrease in permeability and an increase in the hydraulic tortuosity.

A novel linear correlation between the FH and CH permeability as a function of the mean pore size was defined, allowing for the permeability values of a particular test method (e.g. CH) to be determined from another test method (e.g. FH) without requiring both of the time consuming experimentations to be conducted. This correlation is applicable to all GS across different hydraulic gradients and is not limited to the specific materials and results obtained in this study, offering valuable insights into the behaviour of porous media such as permeable concrete. The developed numerical model in this study can be used for permeable concretes of higher porosity with future studies exploring alternative shapes or packing configurations to enhance the model applicability to all permeable concrete structures. Overall, the present study advances the field by presenting a high fidelity, experimentally validated CFD model for investigating water flow through permeable concrete modelled as a packed GS bed. It introduces new insights into the effects of HG, develops a generalised permeability correlation and directly computes hydraulic tortuosity from CFD velocity fields, all while using a realistic, non-overlapping random packing method. These contributions collectively provide a more accurate, scalable and applicable framework for analysing flow in permeable concretes.

## Data Availability

The datasets generated during and/or analysed during the current study are not available to be shared.

## Footnotes

1 Blind pore spaces allow fluid to enter but not exit, as they are only open on one side.
